# The Effectiveness of Simulation in Education 4.0: Application in a Transesophageal Echocardiography Training Program in Malaysia

**DOI:** 10.3389/fsurg.2022.749092

**Published:** 2022-04-04

**Authors:** Sakinah AwangHarun, Noorjahan Haneem Md Hashim, Suhaini Kadiman

**Affiliations:** ^1^IJN College, National Heart Institute, Kuala Lumpur, Malaysia; ^2^Department of Anesthesiology, Faculty of Medicine, Universiti Malaya, Kuala Lumpur, Malaysia; ^3^Department of Anesthesiology and Intensive Care, National Heart Institute, Kuala Lumpur, Malaysia

**Keywords:** simulation as pedagogical tool, simulation in TEE, Kirkpatrick's framework for program evaluation, education 4.0, technology-enhanced learning

## Abstract

**Introduction:**

A Malaysian Higher Education Provider has applied technology as part of its pedagogical approach, in alignment with Education 4. 0. The use of simulation, which aligns with the principles of Education 4.0, employs digital technologies and supports learning by bridging the classroom and the clinical areas. We reported the effectiveness of learning in our program that utilizes multimodal pedagogy, including interactive lectures, pre-recorded video lectures, simulation, and hands-on supervised clinical sessions, using the program's cumulative assessment data.

**Methodology:**

This program evaluation was based on Kirkpatrick's framework. End-points for learning (Kirkpatrick level 2) were analyzed based on improved overall post-test theoretical and clinical assessment performance. Quantitative data analysis of theoretical pre-test, theoretical post-test, clinical assessment, and post-test scores was performed to compare cohorts.

**Results:**

The performance of 19 trainees, over six cohorts from 2012 to 2019, were analyzed. All our trainees had equal opportunities to learn using the multimodal pedagogy, including a simulator. The analysis of pre- and post-theoretical test scores showed a significant improvement in the mean scores (pre-test 48.7% (± *SD* 9), post-test 64.1% (± SD11.5); *p* ≤ 0.001). Overall, 19 out of 21 trainees completed the clinical assessment and case presentation satisfactorily

**Conclusion:**

The Kirkpatrick framework served as a useful framework to perform the evaluation of the TEE program. The significant improvement in post-test scores, when compared with pre-test scores, suggested that the program is effective with regard to learning. As part of a multimodal pedagogy, simulation has proven to be an added value to our training program, and this was reflected by the improvement in the clinical assessment scores when compared to the pre-test scores. This result aligned with the concept of technology-enhanced learning in Education 4.0, where simulation in TEE training is applicable in the Malaysian context.

## Introduction

Education 4.0 is the revolution that focuses on smart technology, including advanced medical devices, artificial intelligence, and robotics, that has given impact to current perioperative cardiothoracic surgical care. Transesophageal echocardiography (TEE) is a high technology device utilized in cardiothoracic surgery to inform the diagnosis and clinical management of patients with diverse pathologies (1). In performing peri-operative TEE, training is necessary to ensure minimal competencies are achieved to provide better clinical outcomes for patients. Due to limited patient access and high-risk patient groups, the use of simulation in TEE training assists the development of the practitioners' competence while maintaining patient safety (2).

Technological advancement transforms teaching and learning strategies in advanced or specialty training (3, 4). In our center, we have applied the principles of education 4.0 in introducing technology and simulation when developing a curriculum for Perioperative Transesophageal Echocardiography.

The use of simulation, which aligns with the principles of Education 4.0, employs digital technologies and supports learning by bridging the classroom and the clinical areas (2, 5). The features of Education 4.0, including personalized learning, learning tools customized to the learner's choice, project-based learning, hands-on and experiential learning, formative and workplace-based assessment, learners' feedback in shaping the curriculum, and independent learning while the teacher facilitates, are all applicable to our TEE program. This is because learners are provided with multiple learning tools to be adaptive to the mentioned features, such as lectures, simulation-based learning tasks, hands-on experience on real patients in the clinical workplace, and trainee evaluation of the program that advises improvement measures. Simulation offers an opportunity for deliberate practice in preparation for hands-on learning in the clinical setting. Knowledge and skills (cognitive and psychomotor) were developed by repetitive and focused training in an alternative safe environment without compromising trainee safety and patient care in the busy and high-risk clinical setting.

The purpose of this study was to measure the effectiveness of a postgraduate TEE training program in Malaysia using the Kirkpatrick Evaluation Model (6). In this study, TEE simulation was used by novice TEE users to learn cognitive and psychomotor skills. We report the effectiveness of learning in our program that utilizes multimodal pedagogy, including interactive lectures, pre-recorded video lectures, simulation, and hands-on supervised clinical sessions, using the program's cumulative assessment data. The Kirkpatrick model was chosen due to its practicality in program evaluation since the information analyzed can be used to improve learners' reaction, learning, behavioral changes, and impact on the institution (6).

## Research Objective

This study aimed to measure the effectiveness of the TEE training curriculum, which included simulation-assisted learning, in a postgraduate TEE training program in Malaysia using the Kirkpatrick evaluation model.

### Research Question

This study mainly asked, what is the effectiveness of the TEE training curriculum, which includes simulation-assisted learning, in a postgraduate TEE training program in Malaysia using the Kirkpatrick education model?

### Study Rationale

The rationale of this study was to improve the effectiveness of the program based on Kirkpatrick's evaluation model (Kirkpatrick level 2: learning).

## Methodology

### Program Description

This program focused on the basic understanding of TEE and its peri-operative applications in cardiothoracic surgical care. This includes the basic understandings of applied ultrasound principles, knobology, clinical indications for TEE, the value of TEE in various clinical situations, understanding standard TEE images, and interpretation of various clinical conditions using TEE over a duration of 7 months to 2 years.

The training was divided into two parts. The first part comprised sessions that include the theoretical knowledge, cognitive and psychomotor skills to acquire and perform semi-quantitative analysis of 20 tomographic views in (i) a simulator and on (ii) 25 patients in Institut Jantung Negara (IJN). Upon achieving satisfactory knowledge and skills in a structured assessment, trainees then proceed to the second part of the program. The second part required trainees to perform and document TEE examination reports on 150 patients at their own institution, over a period of 6 to 18 months.

**Figure 1:**
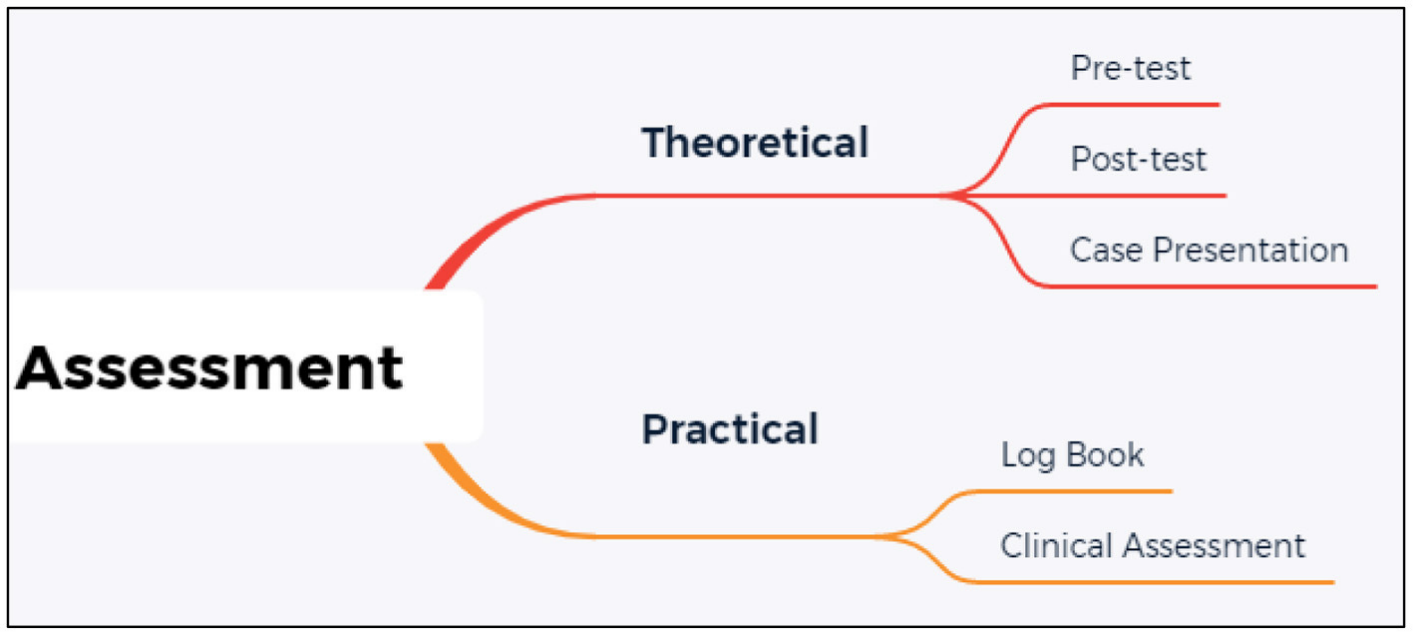
Assessment strategies.

Trainees' knowledge and skills were assessed at four different points throughout the program, strategies summarized in Figure 1. Despite each cohort having the chance of being asked different questions at each assessment session, the domains to be examined and examination methods remained constant for each cohort. Program assessors are internationally certified TEE practitioners and trainers who actively perform perioperative TEE.

Theoretical knowledge and cognitive skills were assessed at the beginning of the program, as a multiple-choice questions pre-test, and at the end of part 1 *via* multiple-choice questions post-test and incorporated in the clinical assessment. The 100 post-test MCQs may differ amongst the cohorts, as the question bank consists of 1500 vetted questions that are blueprinted to the course outcomes. The psychomotor skills were assessed at the end of part 1. At the end of the program, trainees' knowledge, cognitive and psychomotor skills were assessed based on the submitted logbook and a case presentation session. In the case presentation session, trainees were expected to present four cases from their logbooks based on a pre-determined criterion (adult valvular surgery, coronary arterial bypass surgery, combined surgery, and pediatric surgery) supported by its prerecorded relevant echocardiography video loops. The loops were recorded to demonstrate the trainees' understanding of the pathology and their cognitive and psychomotor skills to obtain the relevant views. The question-and-answer session during the presentation assesses the trainees' theoretical understanding.

The pieces of evidence collected from these assessments support that the trainees have achieved the learning outcomes of the program and will be used to measure the effectiveness of the program based on Kirkpatrick Level 2. The description of the program, which includes teaching and learning activities, strategies, and assessment, is summarized in Table 1.

**Table 1:** Summary of teaching and learning activities and strategies and assessment.

**Domains**	**Details**	**Learning activities**	**Study duration (h)**	**Assessment strategies**
Cognitive	Part 1			
	Learning outcomes			
	1. Theoretical knowledge	Lectures (didactic and interactive)	40	100 SBA (MCQ)
	2. Cognitive skills: diagnostic skills	Lectures (interactive); Simulation		
Psychomotor	Learning outcomes			
	3. Technical skills: 3.1. Probe & equipment handling 3.2. Image acquisition 3.3. Image interpretation 3.4. Image storage &retrieval 3.5. Reporting and documentation	Simulation Clinical placement with patient under supervision, to apply the theory, cognitive and psychomotor skills, into appropriate clinical decision making	14	Self-assessment Clinical assessment
Cognitive	Learning outcomes		42	
psychomotor and affective	4. Non-technical skills: 4.1. Teamwork 4.2. Communication 4.3. Situational awareness 4.4. Recognizing limitations	Clinical placement with real patients under supervision, to apply the cognitive and psychomotor skills in clinical environment		Clinical assessment
Cognitive	Part 2			
	Learning outcomes			
	1. Cognitive skills: diagnostic skills			
Psychomotor	Learning outcomes			
	2. Technical skills: 2.1. Probe & equipment handling 2.2. Image acquisition 2.3. Image interpretation 2.4. Image storage &retrieval 2.5. Reporting and documentation	Clinical placement with real patient in respective institution to apply theory, cognitive and psychomotor skills, into clinical decision making in the real clinical environment.	809	Log book; Case presentation
Cognitive	Learning outcomes			
psychomotor and affective	3. Non-technical skills: 3.1. Teamwork 3.2. Communication 3.3. Situational awareness 3.4. Recognizing limitations			

*SBA, Single best answer; MCQ, Multiple Choice Questions*.

**Figure 2:**
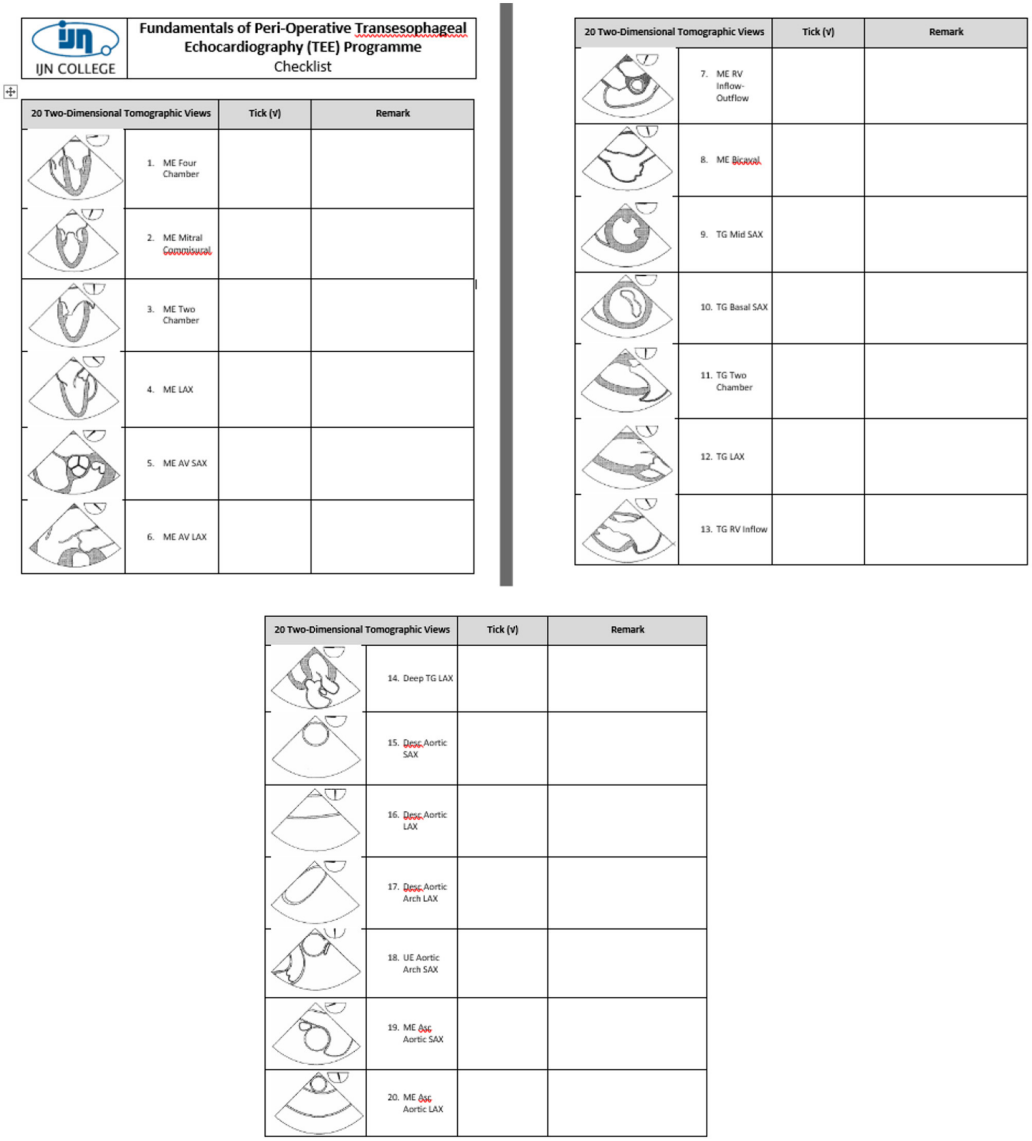
A checklist to guide the image acquisition of the 20 tomographic views in Cohort 2/2012.

**Figure 3:**
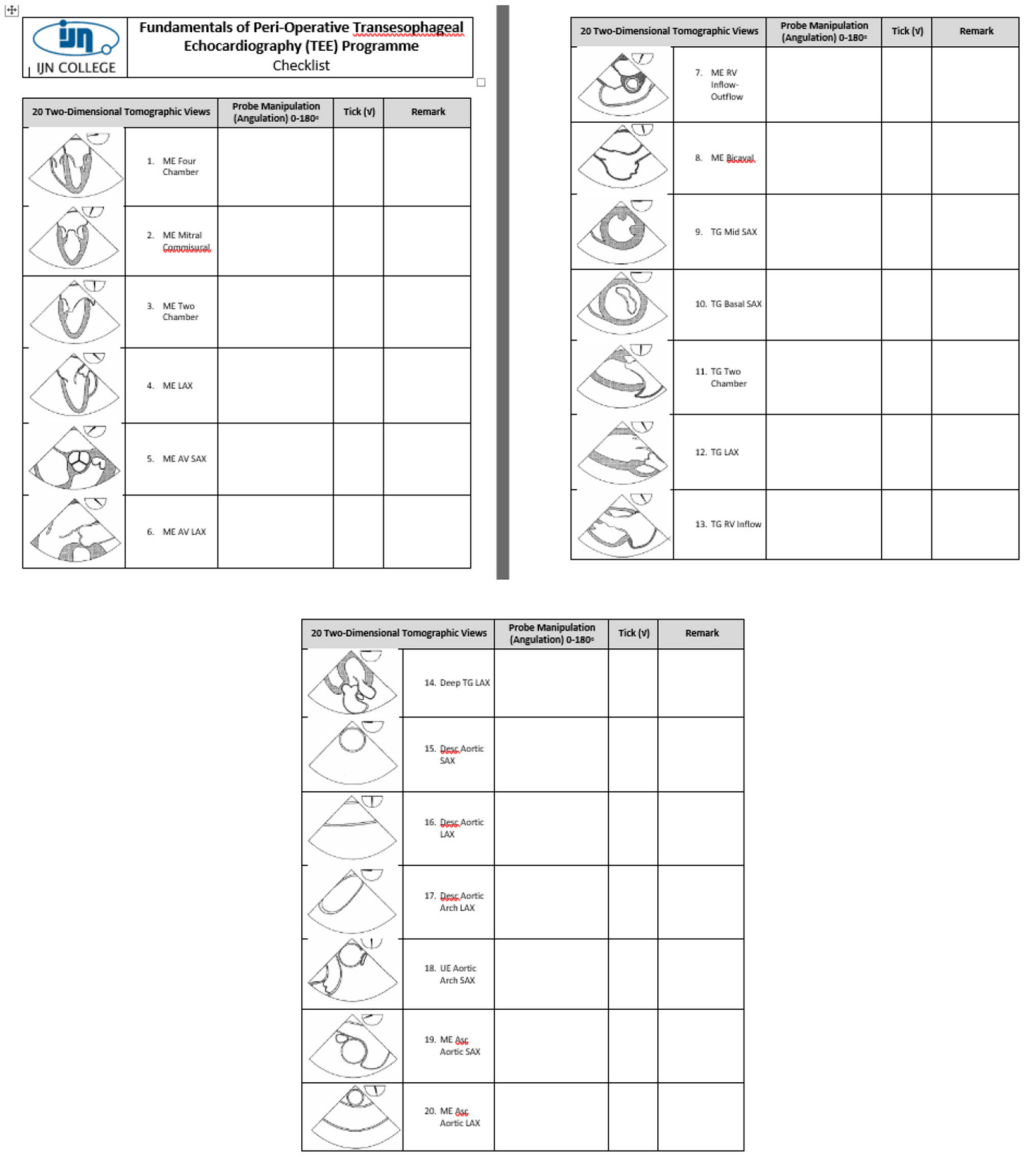
Contents of the checklist include the thematic image of the tomographic views with the appropriate probe position and angulation.

**Figure 4:**
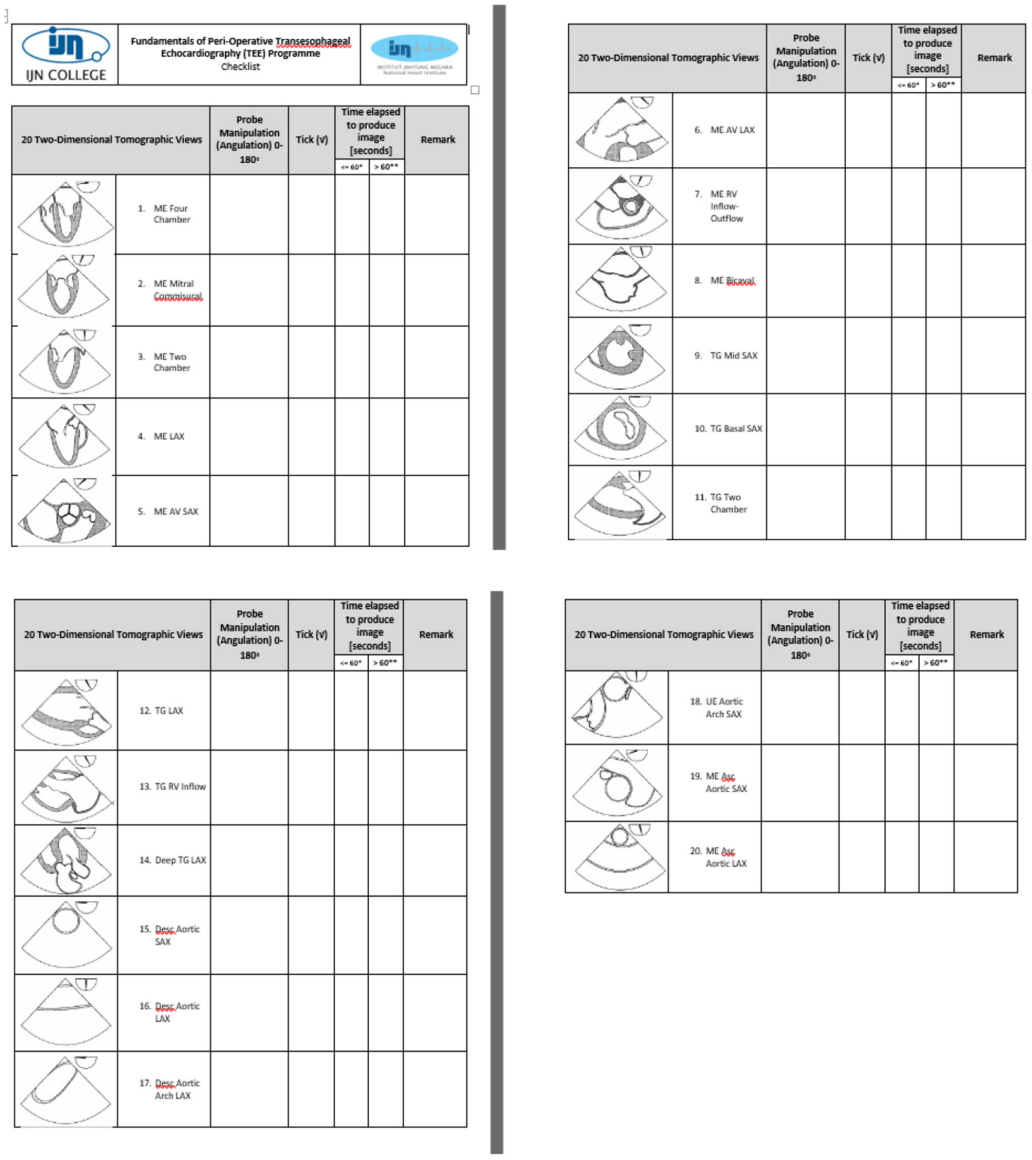
The element of time in the learning outcome of all 20 views: acquisition within 20 min for the fourth cohort in 2016.

The program was evaluated before each intake in accordance with institutional and licensing regulations. Improvements were made according to the trainee, trainer, and other stakeholders' feedback. The simulation was integrated into the curriculum for the second cohort in 2012, based on the feedback from Cohort 1, for improvement of cognitive and psychomotor skills. Changes that were made by applying simulation principles, that include feedback, deliberate practice, and independent learning, were the major contributing factors to learning. A checklist of the 20 tomographic images to be acquired was provided to the trainees, to facilitate image recognition, as shown in Figure 2. The second change made was to improvise the checklist to guide the image acquisition of the 20 tomographic views in 2013 for the third cohort. Contents of the checklist include the thematic image of the tomographic views with the appropriate probe position and angulation, as shown in Figure 3. The third change introduced was to include the element of time in the learning outcome of all 20 views, wherein acquisition was made within 20 mins for all 20 basic views for the fourth cohort in 2016, as shown in Figure 4. The time limit chosen was based on recorded time to image acquisition of the previous batches both on the simulator and patients. The fourth change was to provide personalized on-demand coaching to acquire optimal image within 2 min for the same cohort while trainees learn on the TEE simulator.

### Study Sample

The study sample included anesthesiologists, cardiothoracic anesthesiologists, cardiologists, and intensivists who enrolled in the Perioperative Transesophageal Echocardiography program at Institut Jantung Negara from 2012 to 2019. Sampling was universal, as all participant's performances were included in the analysis.

The rationale for universal sampling is perioperative TEE is a niche area focusing on the perioperative care of cardiac patients, and the pool for potential participants is small, to begin with. In addition, the limited number of learners enrolled in the program was also limited by our aim to maintain an optimal trainer: trainee ratio and procedure: trainee ratio to ensure each learner receives an equal learning opportunity.

The heterogeneity and possible differences between trainees on joining the program are expected of professionals and adult learners. However, all enrolled trainees fulfilled the minimum program entry requirement of being licensed physicians enrolled or completed their specialty training program. Their baseline knowledge and cognitive skills reflecting prior learning are assessed using the theoretical pre-test upon entry. Their psychomotor skills were not assessed.

### Outcome Measures

The outcomes studied were based on the Kirkpatrick program evaluation framework, learning (Kirkpatrick level 2) (6). Overall learning was defined as improved theoretical post-test results and clinical assessment performance for all trainees. Cohort learning, to compare the learning effects of pedagogical interventions, was defined as improved theoretical post-test results, clinical assessment, and case presentation scores between cohorts.

### Data Analysis

Retrospective data were analyzed using IBM SPSS version 27 (IBM Corp, Armonk, NY, USA). Paired sample *t*-tests were used to compare means where data was normally distributed, for all cohorts. When comparing means between cohorts, no statistical analysis was performed due to the small sample size.

## Results

The performance of 19 trainees, over six cohorts from Cohort 2/2012 to 8/2019 were analyzed. Trainees from Cohort 1 were excluded from the analysis as they were the pilot group for the program and had different pedagogy and assessment time points. All trainees analyzed had equal opportunities learning using the multimodal pedagogy, including a simulator. The summary of trainee scores for all assessment components is shown in Table 2.

**Table 2:** Summary of assessment scores of all the trainees, with changes in pedagogy.

**Cohort**	**Year**	**Changes**	**Number of trainees**	**Mean theory scores (%, ±SD)**		**Changes in scores (post-test- pre-test)**	**Mean clinical assessment scores (%, ±SD)**	**Mean logbook scores (%)**	**Mean case presentation score (%, ±SD)**
				**Pre-test**	**Post-test**				
1	2012	-	2	-	74.5	-	59.5 (12.73)	100	80 90.00)
2	2012	Simulation and checklist	3	45.3	55.3	+10	68.7 (11.02)	100	64.5 (3.54)
3	2014	Checklist with probe position and angulation	2	49.5	57.5	+8	66.0 (22.62)	100	69
4	2016	Time + coaching	2	62.0	68.5	+6.5	75.5 (7,78)	100	78
5	2016	-	2	48.0	60.5	+12.5	65.5 (0.71)	100	63.5 (0.71)
6	2017	-	3	50.0	66.3	+16.3	61.0 (6.56)	100	75.5 (4.77)
7	2018	-	4	44.5	69.25	+24.75	70.0 (14.14)	100	83 (15.56)
8	2019	-	3	47.3	76.0	+28.7	Not applicable	100	Not applicable

**Figure 5:**
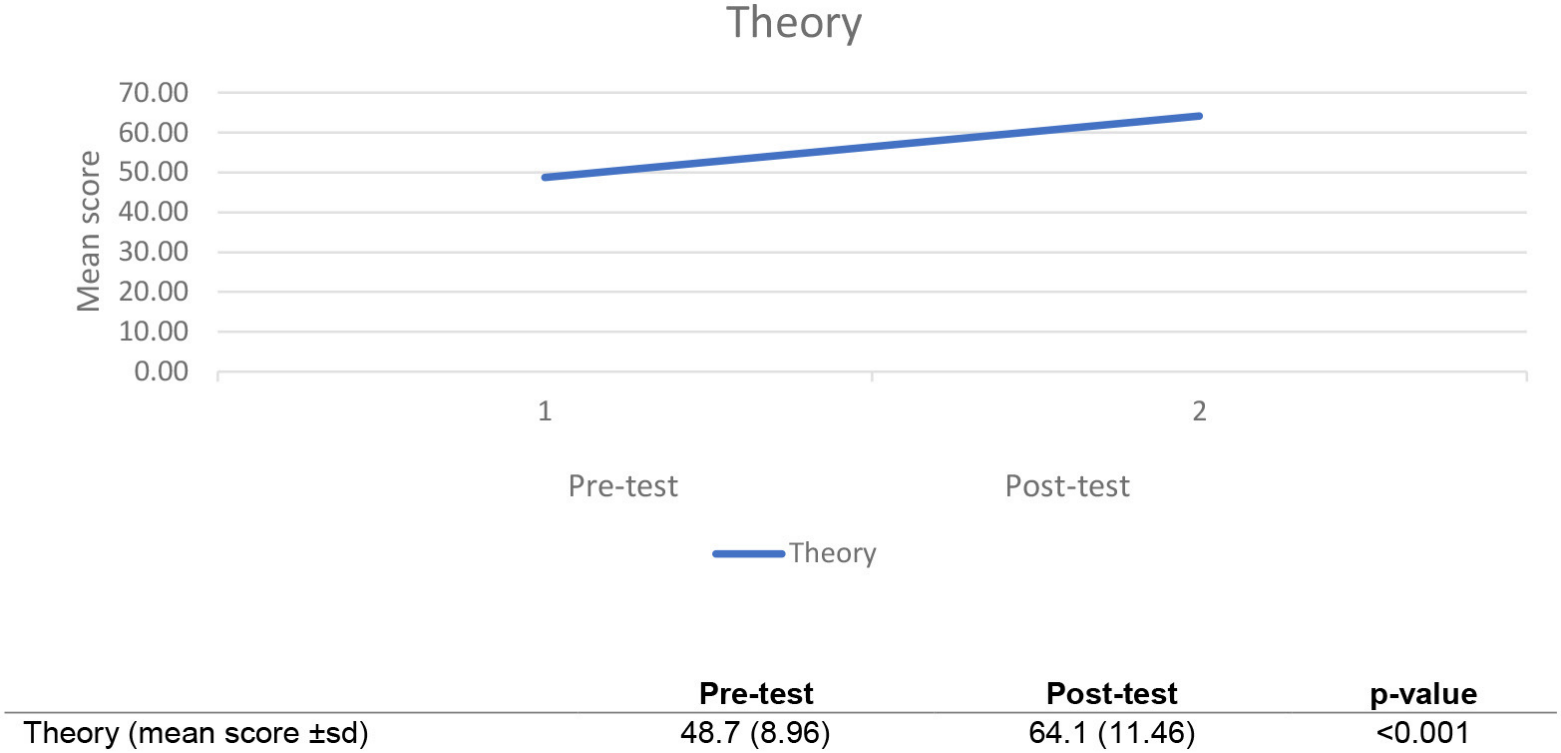
Chart showing improvement in post-tests mean scores across cohorts.

The performances of 19 trainees were analyzed for the theory assessment component. The analysis of pre- and post- theoretical test scores showed a significant improvement in the mean scores (pre-test 48.7 % (± *SD* 9), post-test 64.1% (± *SD* 11.5); *p* ≤ 0.001), shown in Figure 5.

Overall, 19 out of 21 trainees completed the clinical assessment. The means for each change in program delivery improvements were 66% (introduction of checklist with probe position and angulation) and 67.2% (time as part of learning outcome and personalized on-demand coaching) respectively compared to the original program delivery method of 59.5%.

## Discussion

Curriculum integration in simulation-based healthcare education allows education providers to perform a goal-directed evaluation of student learning and supports sustained use of its technology (3). When the technology became available, its use was piloted in our program and was formally integrated into the curriculum based on learner feedback for the second cohort. There was an improvement in the mean clinical performance scores (59 and 67.8%) in the cohorts pre- and post-integration of simulation. This is supported by Vega (7). where the effectiveness of simulation training is enhanced by verbal cues, training sequences (evidenced by the use of checklist), visual cueing from the checklist, and feedback from high fidelity simulator.

The use of simulation in education has been implemented in other TEE training programs. Simulation technology has been available for many decades and has been shown to improve learning and patient outcomes in many fields (8). TEE simulation has been used to train cognitive, psychomotor, and affective domains that increase performance. The simulation is also capable of reducing incidences of human error and increasing the standards and quality of patient care. This supports the findings in our program, where trainees were able to demonstrate the 20 tomographic image acquisition within 20 min by practicing with simulation, aided by a holistic checklist and personalized coaching and feedback.

The significant improvement of the theoretical post-test, when compared to the pre-test, shows that learning occurred during the program. Though the best method would be to include a comparison of pre- and post-simulation clinical performance, this was neither feasible nor safe in our setting. Nazerian et al. agreed to evaluate the ability of emergency physicians (EPs) to obtain and maintain skills in performing TEE to aid resuscitation after a course with clinical training in the cardiac surgical theater (1). Therefore, training on a simulator improves competency and reduces errors and harm to patients. Training using the simulation for TEE experience may improve proficiency, learning speed, increase trainees' comfort, and increase performance while performing the procedure in clinical practice after completion of the study. In this study, the program allocated 14 h for TEE simulation training for trainees to practice before placement in the clinical environment. The TEE simulator created a dynamic environment in which trainees can improve their abilities in critical thinking, decision making, and clinical judgment. Within the structure of simulation, trainees focus on psychomotor components to master the TEE probe manipulation skills to obtain images by using TEE technology. A variety of TEE simulator models has been incorporated into our curriculum to enhance the efficiency of teaching and evaluate proficiency in TEE.

Incorporating simulation, either as a pedagogical or assessment tool, was also documented by two programs that train physicians in focused cardiac ultrasound for the emergency setting. The first program focused on training residents in French cardiology, anesthesiology, and emergency medicine setting (9). The investigators described a three-stage training program, which included didactic lectures in an e-learning platform, free access to a TTE simulator with on-demand experts for one day, followed by placement in the echocardiography laboratory where the trainees perform focused TTE in 10 consecutive patients. The third stage was self-training in image analysis in an online platform. All trainees underwent a pre-test evaluation using TEE simulators, using the same criteria as the post-test evaluation and end of stage 2 assessment. Trainees' cognitive (ability to interpret images) and practical skills were assessed using simulators at the end of the first stage and with real patients during the second stage. The time to image acquisition was recorded. TEE examinations were recorded and assessed by two blinded assessors. Image quality, visualization of structures, image stability, and interpretability of the TEE examinations were the criteria being scored. The final evaluation was an interpretation of 20 video clips of the TEE examination. The results of this study showed significant improvement in practical and interpretive skills of trainees at the end of echocardiography laboratory placement. The main difference with our program is their pre-test assessment strategy using simulation, where baseline image acquisition skills were recorded and compared to after training, and the images were recorded and scored by 2 different assessors. This allows tracking of trainees' performance, and the program would be able to identify trainees' learning curves and areas for improvement.

In another study in North America, Beraud et al. (10) also designed and implemented a focused TTE curriculum, for critical care medicine fellows. The training consisted of weekly lectures and 15-h of one-on-one bedside ultrasound scanning instructions in the ICU by a cardiologist instructor. Trainees then performed, recorded, and reported TEE examinations, which were assessed by the cardiologist instructor. Feedback was provided regarding image quality and interpretation. At the end of 1 year, trainees were assessed using the recording of their last three examinations and using a simulator. The results show significant improvement in the scanning and diagnostic abilities of the fellows. This shows that simulation, when combined with real patient experience can be an effective tool for learning echocardiography.

The significant improvement of the theoretical post-test and practical clinical shows that learning occurred during the program. Though the best method would be to compare pre- and post-clinical performance, this was neither feasible nor safe in our setting. Therefore, theoretical components, especially related to diagnosis were incorporated in the clinical performance assessment.

The advancement of technology has been changing significantly, especially in cardiovascular and thoracic services as well as in the education industry. These technological advancements, along with changing expectations regarding training hours, patient safety, and limited patient encounters, have supported the paradigm of incorporating technology in providing education (3). Integrating simulation as a pedagogical tool is the best approach to link theoretical knowledge, workplace-based learning, and clinical practice (4). The TEE simulator as an advanced technology plays an important role in helping trainees become active learners. Learning and mastery are evidenced in our program with the improvement in theoretical post-tests, compared to pre-test, ability to acquire, interpret and report images in real patients in the authentic clinical setting (11). The TEE simulator technology has created new and diverse methods for conducting these processes, supported the trainees in multiple ways. Technology integration is the use of technology tools in general content areas in education to allow trainees to apply TEE simulator and technology skills to learning and problem solving, critical thinking, decision making, and clinical judgment (12).

Kirkpatrick's framework for program evaluation was chosen, as it provided a clear taxonomy for decision-making (13). We chose it for its simplicity and helped us focus on the learners: their reactions, learning, behavioral change, and system-level impact. We chose to focus on learning at this point as we wanted to learn the immediate effects of our educational interventions.

Our study has a few limitations. Our sample size was very limited. As mentioned previously, this was due to the limited pool of learners as this is a very niche learning area, as well as due to our aim to ensure that opportunities for learning were constant and equal for all learners.

We were also not able to provide direct supervision of the first 50 TEE performance and interpretation, as recommended by the 2013 consensus statement of the American Society of Echocardiography and the Society of Cardiovascular Anesthesiologists basic TEE training requirement (14) due to the limited number of patients in our center and our learners are only allowed to be in our institution for one month.

The significant improvement measured in the theoretical post-test and clinical assessment shows that learning occurred during the program. Though the best method would be to compare pre- and post-simulation clinical performance, this was neither feasible nor safe in our setting. Therefore, theoretical components, especially related to diagnosis were incorporated in the clinical performance assessment.

Another limitation was we were not able to cross-check self-directed learning TEE findings in part 2, as trainees were in their own institutions, and it was not feasible to record all TEE examination findings due to potential patient confidentiality issues and unavailability of assigned trainers in the respective centers. When crosscheck was available, it was due to chance, rather than, designed in the curriculum.

Despite these limitations along with the results only showing statistical significance in the theory components, the experiences reported by the trainees that were paired with their performance had provided meaningful feedback for program improvement to support learning.

## Future Recommendations

Performance of TEE requires the psychomotor ability to obtain interpretable echocardiography images and simulation is an effective tool for learning psychomotor skills by novices. The COVID-19 pandemic opens other opportunities for the application of technology into online and distant learning. This can be facilitated with a learning management system and a virtual learning environment. Integrating a learning management system that supports word processing documents, audio and video streaming into our program would facilitate multiple teaching, learning, and assessment activities: access to lectures, remote case presentations, remote preceptors and facilitators, and theoretical and virtual live cases for assessments. Though the advantages of online learning have been highlighted in numerous studies, items to be integrated into the LMS must be carefully selected. Items that can be selected are videos, online discussions, and case presentation items. Novel items that can be included are remote clinical teaching and assessment. The LMS can also be used to track trainee performance on the simulators. This would facilitate trainers and trainees to decide trainee readiness for practice in real patients.

## Conclusion

Integrating a TEE simulator as a pedagogical tool for our postgraduate TEE training program has proven to be effective in supporting learning theoretical knowledge and clinical competency in perioperative cardiac surgical TEE amongst our trainees.

The TEE Simulator was helpful in providing a bridge from theory to the clinical environment by supporting trainees' cognitive and psychomotor skills.

The Kirkpatrick framework served as a useful framework to evaluate the effectiveness of this training program. The significant improvement in post-test scores, when compared with pre-test scores, suggested that the program is effective with regards to learning. Simulation, as part of a multimodal pedagogy, has added value to our training program, and this was reflected by improvement in the clinical assessment scores when compared to the pre-test scores. This result aligned with the concept of technology-enhanced learning in Education 4.0, where simulation in TEE training is applicable in the Malaysian context.

## Data Availability Statement

The original contributions presented in the study are included in the article/supplementary material, further inquiries can be directed to the corresponding author.

## Author Contributions

SA: program design and developer and implementation, contributed to design, conception of the study (Introduction, Methodology, Results, Discussion, and Future Recommendation), the acquisition of data, analysis, interpretation, and wrote the paper. NM: analysis and interpretation, wrote the paper (Introduction, Methodology, Results, and Conclusion), and performed first and second proofreading. SK: contributed to program design and implementation, first, and second proofreading. All authors contributed to the article and approved the submitted version.

## Conflict of Interest

The authors declare that the research was conducted in the absence of any commercial or financial relationships that could be construed as a potential conflict of interest.

## Publisher's Note

All claims expressed in this article are solely those of the authors and do not necessarily represent those of their affiliated organizations, or those of the publisher, the editors and the reviewers. Any product that may be evaluated in this article, or claim that may be made by its manufacturer, is not guaranteed or endorsed by the publisher.
